# Efficacy of apatinib 250 mg combined with chemotherapy in patients with pretreated advanced breast cancer in a real-world setting

**DOI:** 10.3389/fonc.2023.1076469

**Published:** 2023-06-16

**Authors:** Ruyan Zhang, Yifei Chen, Xiaoran Liu, Xinyu Gui, Anjie Zhu, Hanfang Jiang, Bin Shao, Xu Liang, Ying Yan, Jiayang Zhang, Guohong Song, Huiping Li

**Affiliations:** Key Laboratory of Carcinogenesis and Translational Research (Ministry of Education/Beijing), Department of Breast Oncology, Peking University Cancer Hospital and Institute, Beijing, China

**Keywords:** breast cancer, apatinib, chemotherapy, efficacy, safety

## Abstract

**Objectives:**

This study evaluated the efficacy and safety of apatinib (an oral small-molecule tyrosine kinase inhibitor targeting VEGFR-2) 250 mg combined with chemotherapy in patients with pretreated metastatic breast cancer in a real-world setting.

**Patients and methods:**

A database of patients with advanced breast cancer who received apatinib between December 2016 and December 2019 in our institution was reviewed, and patients who received apatinib combined with chemotherapy were included. Progression-free survival (PFS), overall survival (OS), the objective response rate (ORR), the disease control rate (DCR), and treatment-related toxicity were analyzed.

**Results:**

In total, 52 evaluated patients with metastatic breast cancer previously exposed to anthracyclines or taxanes who received apatinib 250 mg combined with chemotherapy were enrolled in this study. Median PFS and OS were 4.8 (95% confidence interval [CI] = 3.2–6.4) and 15.4 months (95% CI = 9.2–21.6), respectively. The ORR and DCR were 25% and 86.5%, respectively. Median PFS for the previous line of treatment was 2.1 months (95% CI = 0.65–3.6), which was significantly shorter than that for the apatinib–chemotherapy combination (p < 0.001). No significant difference was identified in the ORR and PFS among the subgroups(subtypes, target lesion, combined regimens and treatment lines). The common toxicities related to apatinib were hypertension, hand-foot syndrome, proteinuria, and fatigue events.

**Conclusion:**

Apatinib 250 mg combined with chemotherapy provided favorable efficacy in patients with pretreated metastatic breast cancer regardless of molecular types and treatment lines. The toxicities of the regimen were well tolerated and manageable. This regimen could be a potential treatment option in patients with refractory pretreated metastatic breast cancers.

## 1 Introduction

Metastatic breast cancer (MBC) remains an incurable disease, with median overall survival (OS) of about 3 years and a 5-year survival rate of around 25%, even in countries without medicine availability problems (1).

Novel therapeutic strategies for MBC have been established in recent years. Cyclin-dependent kinase 4 and 6 (CDK4/6) inhibitors for hormone receptor(HR) positive/human epidermal growth factor 2 (HER2)-negative MBC, trastuzumab emtansine(T-DM1) and T-Dxd for HER2 positive MBC, immune check point inhibitor pembrolizumab and sacituzumab govitecan (SG) for metastatic triple negative breast cancer(TNBC), and poly(ADP ribose) polymerase (PARP) inhibitors for HER2 negative MBC with germline BRCA1/2 mutation have become the standard treatment recommended by guidelines. However, the first CDK4/6 inhibitor palbociclib and T-DM1 were approved by China Food and Drug Administration(CFDA) in August 2018 and January 2020 respectively, and they were not included in medical insurance until March 2023; Until today, pembrolizumab and olaparib have not been approved by CFDA for the treatment of metastatic breast cancer, pembrolizumab was approved only for treatment of early TNBC with high risk of recurrence in November 2022, olaparib was approved only for ovarian cancer and prostate cancer in China; T-Dxd and SG have not yet launched in Mainland of China until now. So in the real world clinical practice, a considerable number of patients didn’t receive these treatments due to drug accessibility and/or expensive cost which was not covered by local medical insurance. Moreover, some patients who received above treatments did not respond to the therapy, and some who experienced initial response still developed resistance inevitably afterwards. In patients with taxane- and anthracycline-resistant human epidermal growth factor 2 (HER2)-negative MBC, traditional chemotherapy agents including capecitabine, vinorelbine, gemcitabine, or platinum agents are usually considered as treatments of choice. There is no evidence suggesting that any chemotherapeutic drugs have superior efficacy in the second and later lines, and new drugs or strategies are required for this population of patients. Some new therapeutic strategies for MBC such as anti-angiogenesis therapy, androgen receptor antagonists, micro-RNA based therapy, proteolysis targeting chimeric molecules (PROTACs) and others are under exploration,some of them have initially shown potential benefits (2, 3).

Previous researches indicated that angiogenesis is vital for tumor growth and metastasis (4, 5). The vascular endothelial growth factor (VEGF) pathway plays an important role in angiogenesis in cancer (6, 7), and VEGF receptor-2 (VEGFR-2) is the key signaling receptor involved in this pathway (8, 9). Therefore, anti-angiogenesis is an important anti-cancer strategy (7). The anti-VEGF monoclonal antibody bevacizumab, when added to chemotherapy, has been demonstrated to significantly increase progression-free survival (PFS) in patients with metastatic triple-negative breast cancer (TNBC) in the first- and second-line settings (10–13).

Apatinib is an oral small-molecule tyrosine kinase inhibitor (TKI) selectively targeting VEGFR-2, and apatinib monotherapy has been approved by the CFDA for the third-line treatment of gastric cancer based on its remarkable survival benefits (14).Two prospective open-label, multicenter, phase 2 trials preliminarily revealed the satisfying efficacy and acceptable toxicities of apatinib monotherapy in TNBC and non-TNBC (15, 16).

Preclinical studies illustrated that combined treatment with apatinib can improve the efficacy of chemotherapy and reverse chemotherapeutic drug resistance in tumor cells (17) (18–20). Limited studies have explored the efficacy and safety of apatinib combined with chemotherapy in solid tumors including breast cancer, and efficacy and good tolerance were preliminarily observed (21–26).

Based on above results, we performed a retrospective study to further evaluate the efficacy and safety of apatinib combined with chemotherapy in patients with MBC who failed standard treatment in a real-world setting.

## 2 Materials and methods

### 2.1 Methods

A database of patients with breast cancer treated with apatinib combined with chemotherapy from December 2016 to December 2019 in the Department of Breast Oncology of Peking University Cancer Hospital and Beijing Institute of Cancer Prevention was reviewed.

The inclusion criteria were as follows: pathologically confirmed locally advanced breast cancer or MBC; failed previous standard treatments; treated with apatinib combined with chemotherapy, and finished at least one cycle of treatment to permit toxicity evaluations.

The estrogen receptor (ER), progesterone receptor (PgR), and HER2 were recorded. For patients who underwent biopsies of the metastatic sites, the status of these receptors was determined on the basis of the latest pathological test before apatinib treatment. ER/PgR negativity was defined as <1% positive tumor cells with nuclear staining on immunohistochemistry (IHC); a HER2-negative status was defined as an IHC score of 0–1; and negativity was defined by fluorescent *in situ* hybridization in accordance with the American Society of Clinical Oncology guidelines.

Tumor assessments were evaluated every two or three cycles of treatment based on the Response Evaluation Criteria In Solid Tumors (RECIST) (version 1.1). PFS was defined as the time interval from initiating apatinib therapy to disease progression or death, whichever occurred first. OS was considered the interval from initiating apatinib therapy to death from any cause or the last follow-up visit. Adverse events (AEs) were assessed according to the National Cancer Institute Common Terminology Criteria for Adverse Events (version 4.03).

### 2.2 Statistical analysis

Median PFS and OS were calculated using the Kaplan–Meier method, and inter-group comparisons were performed using the log-rank test. Pearson’s χ^2^ test or Fisher’s exact test was used to analyze treatment efficacy. Cox regression analysis was used to analyze the correlations between factors and prognosis. SPSS version 26.0 was used for all statistical analyses, and p < 0.05 was considered statistically significant.

## 3 Results

### 3.1 Patient characteristics

In total, 61 patients with MBC who received apatinib combined with chemotherapy were included. All patients were given apatinib 250 mg per day orally, and chemotherapy was based on physician’s choice. Patient characteristics at baseline were shown in **Table 1**.

**Table 1:** Patient characteristics at baseline.

Characteristics	Number N=61	%
Age		
<60 years	54	88.5
≥60 years	7	11.5
Histology		
Ductal	53	86.9%
Lobular	2	3.3%
Metaplastic carcinoma/phylloides sarcoma	6	9.8
Molecular Subtype		
TNBC	23	37.7
ER positive/Her2 negative breast cancer	32	52.5
HER2 positive breast cancer	6	9.8
Metastatic sites		
Lymph nodes	33	54.1
Chest wall	34	55.7
Bone	25	41
Liver	19	31.1
Lung	16	26.2
Pleural	16	26.2
Brain	2	3.3%
Number of prior chemotherapy lines in metastatic setting, median line=2(0-5)		
0	5	8.2%
1	16	26.2
2	20	32.8
≥3	20	32.8
Combined chemo-regimens		
Gemcitabine	16	26.2
Vinorelbine	16	26.2
Taxanes	15	24.6
Capecitabine	10	16.4
Platinum	7	11.5
Anthracycline	4	6.6
Sequence of chemo and apatinib		
Synchronously	49	80.3
Chemo first	9	14.8
Apatnib first	3	4.9

TNBC, triple-negative breast cancer; ER, Estrogen receptor; HER2, human epidermal growth factor receptor 2.

The median age at the start of apatinib therapy was 49.9 years (range, 31–67 years). Concerning the molecular subtype, 23 patients (37.7%) were diagnosed with TNBC, 32 patients (52.5%) had ER-positive breast cancer, and 6 patients (9.8%) had HER2-positive breast cancer. More than half of the patients had lymph node and chest wall metastasis (54.1% and 55.7%, respectively), 19 patients (31.1%) had liver metastasis, and 16 patients (26.2%) had lung metastasis.

All 61 patients had previously received chemotherapy containing anthracycline or taxane, and 56 patients (91.8%) had received at least one chemotherapeutic regimen for metastatic disease before the use of apatinib.The median number of prior chemotherapy lines was 2 (0–5). Five patients (8.2%) who received apatinib in the first-line setting all had disease-free survival(DFS) shorter than 12 months. Patients with hormone receptor-positive breast breast cancer had received at least one regimen of endocrine treatment. Patients with HER2-positive disease had progressed on previous anti-HER2 therapy. None of the HR+HER2- patients received CDK4/6 inhibitors and only one of them received everolimus before apatinib, one of the five HER2 positive patients received T-DM1 in a phase III clinical trial before apatinib, and none of the TNBC patients received immunotherapy before apatinib. Among all of the 61 patients, one TNBC patient harbored suspected pathogenic mutation of germline BRCA1, one HR+HER2 patient harbored pathogenic mutation of germline BRCA2, neither of them received PARP inhibitor treatment.

The chemotherapies used in combination regimens were gemcitabine (16, 26.2%), vinorelbine (16, 26.2%), taxanes (15, 24.6%), capecitabine (10, 16.4%), platinum (7, 11.5%), and anthracycline (4, 6.6%). Three patients with HER2-positive disease received anti-HER2 targeted therapy (trastuzumab, pyrotinib, and lapatinib, respectively) along with apatinib and chemotherapy.

### 3.2 Efficacy

**Figure 1:**
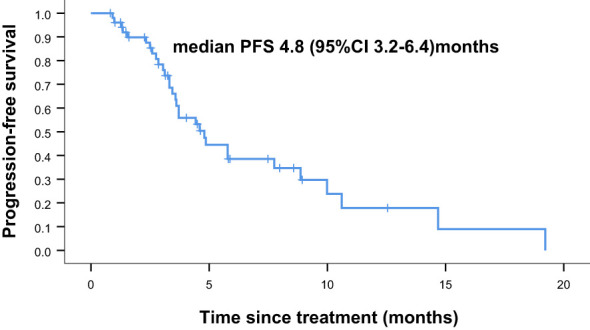
Progression free survival (PFS) of 52 evaluable patients.

**Figure 2:**
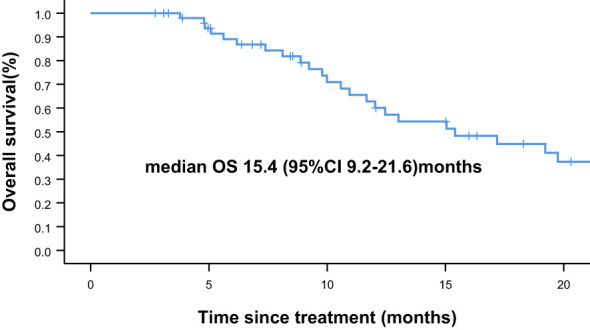
Overall survival (OS) of 52 evaluable patients.

**Figure 3:**
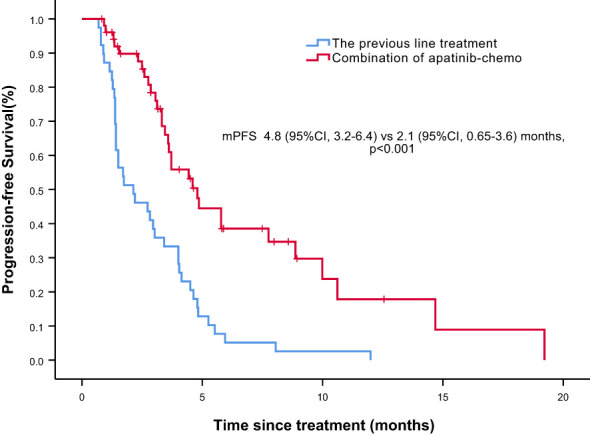
Progression free survival (PFS) of apatinib combined with chemotherapy versus PFS of the previous line treatment.

Overall, nine of the 61 patients required treatment discontinuation in the first two chemotherapy cycles because of intolerable toxicities, and the tumor assessment could not be completed. Therefore, 52 patients were included in the analyses of PFS, OS, and clinical responses. With a median follow−up of 7.4 months (range, 2.4–41.7 months), 31 of 52 patients had progressive disease (PD), and 25 deaths occurred. Median PFS was 4.8 months (95% confidence interval [CI] = 3.2–6.4 months, **Figure 1**: PFS of 52 evaluable patients), and median OS was 15.4 months (95% CI = 9.2–21.6 months, **Figure 2**: OS of 52 evaluable patients). Median PFS for the previous line of treatment (chemotherapy alone) was 2.1 months (95%CI = 0.65–3.6 months), which was significantly shorter than that of apatinib combined with chemotherapy(4.8 months, 95% CI =3.2–6.4 months, p < 0.001), comparisons were performed using the log-rank test (**Figure 3**: PFS of apatinib combined with chemotherapy versus PFS of the previous line treatment.).

**Figure 4:**
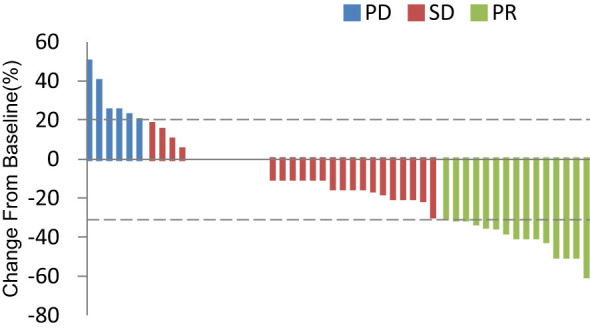
Best overall response of 52 evaluable patients. PD, progressive disease; PR, partial response; SD, stable disease.

**Figure 5:**
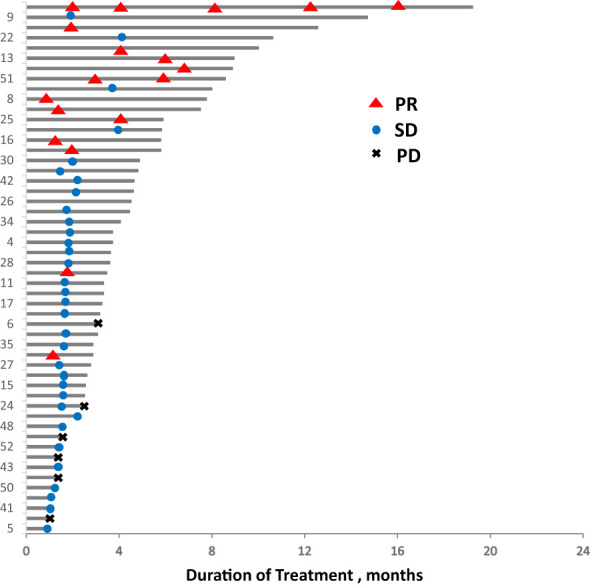
Duration of treatment and response. PD, progressive disease; PR, partial response; SD, stable disease.

In total, 13 (25.0%) and 34 (65.4%) patients had a best clinical response of partial response (PR) and stable disease (SD), respectively, and no patients had complete response (CR). The overall response rate (ORR) was 25.0% (13/52), and the disease control rate (DCR) was 86.5% (45/52, **Table 2**, **Figure 4**. Best overall response of 52 evaluable patients **Figure 5**. Duration of treatment and response).

**Table 2:** Best response in evaluated patients (N=52).

Best response	N (%)	mPFS (months,95%CI)	P value
CR	0	–	
PR	13 (25)	10.0 (7.7-12.2)	<0.001
SD	32 (61.5)	3.7 (3.5-3.9)	
PD	7 (13.5)	1.3 (1.3-1.4)	
ORR (CR+PR)	13 (25)	–	
DCR (CR+PR+SD)	45 (86.5)	–	

CR, complete response; PR, partial response; SD, stable disease; PD, progressive disease; ORR, objective response rate; DCR, disease control rate; mPFS, median progression free survival.

Response and PFS in different subgroups were analyzed as presented in **Table 3**. Median PFS was longer for patients who achieved PR than for those who did not achieve PR (10.0, 3.7, and 1.3 months for the PR, SD, and PD groups, respectively; P < 0.001). The ORR was best in the gemcitabine group (42.9% [6/14]) among all combination regimen groups, and patients for whom the liver or chest wall/lymph nodes were the target lesions displayed satisfying ORRs (33.3% [5/15] and 28.6% [8/28], respectively). Meanwhile, the ORR was 0%(0/5), 18.8%(3/16), and 32.3%(10/31) for the first, second, and third or later lines, respectively, and all the five patients in the first group had a best clinical response of stable disease, and median PFS was similar in different treatment lines. Regarding different molecular types, both the ORR and PFS were worse in the TNBC group than in the ER-positive/HER2-negative and HER2-positive groups. No significant difference was identified in the ORR and PFS among the subgroups by the log-rank test for univariate analysis.

**Table 3:** Response and PFS in different subgroups.

Subgroup	ORR		PFS	
	ORR (N,%)	p value	mPFS (months,95%CI)	p value
Subtypes				
TNBC	2/21 (9.5)	0.081	3.6 (2.1-5.0)	0.184
ER+/HER2-	8/26 (30.8)		5.8 (0.4-11.1)	
HER2+	3/5 (60.0)		7.8 (3.5-12.0)	
Target lesion				
Liver	5/15 (33.3)	0.492	3.7 (0.0-9.0)	0.988
Chest wall/LN	8/28 (28.6)		5.8 (3.0-8.6)	
Lung	0/6 (0)		3.3 (-)	
Others	0/3 (0)		4.8 (-)	
Combined regimen				
Gemcitabine	6/14 (42.9)	0.422	5.8 (2.8-8.8)	0.805
Vinorelbine	3/13 (23.1)		3.6 (2.6-4.6)	
Capecitabine	1/6 (16.7)		19.2 (-)	
Taxanes	2/10 (20.0)		4.8 (4.2-5.4)	
Others	1/9 (11.1)		4.6 (3.4-5.8)	
Treatment line				
1	0/5 (0)	0.407	4.8 (4.4-5.2)	0.655
2	3/16 (18.8)		4.4 (3.0-5.9)	
≥3	10/31 (32.3)		4.9 (2.9-6.8)	

ORR, objective response rate; mPFS, median progression free survival; LN, lymph node.

### 3.3 Safety

A total of 61 patients were analyzed for toxicity. Nine patients discontinued the combination treatment in the first two cycles because of intolerable toxicities which including hypertension (four cases), thrombocytopenia (three cases), fever(one case), anorexia(one case).The most common non-hematologic AEs were hypertension, hand-foot syndrome, proteinuria, fatigue, liver dysfunction and anorexia, whereas hematologic AEs, including neutropenia, anemia and thrombocytopenia, occurred at high rates because of the use of combination therapy (**Table 4**). Most toxicities were generally grade 1–2 and manageable.

**Table 4:** Adverse Events graded based on CTCAE 4.0.

Adverse events	Grade1-2 (n,%)	Grade3-4 (n,%)	All grades (n,%)
Hypertention	14 (23.0)	2 (3.3)	16 (26.0)
Hand-foot syndrome	12 (19.7)	3 (4.9)	15 (24.6)
Proteinuria	10 (16.4)	2 (3.3)	12 (19.7)
Fatigue	10 (16.4)	0	10 (16.4)
Anorexia	9 (14.8)	0	9 (14.8)
Neutropenia	25 (41.0)	3 (4.9)	28 (45.9)
Anemia	25 (41.0)	3 (4.9)	28 (45.9)
Thrombocytopenia	10 (16.4)	2 (3.3)	12 (19.7)
Liver dysfunction	9 (14.8)	1 (1.6)	10 (16.4)

## 4 Discussion

Our present study reported the efficacy and safety of apatinib combined with chemotherapy in patients with pretreated MBC in a real world setting. In this study, all 52 patients were resistant to standard treatment, the median number of prior chemotherapy lines was 2(0-5), and the cohort included patients with TNBC and ER-positive/HER2-negative breast cancer with resistance to chemotherapy and endocrine therapy and patients with HER2-positive breast cancer who progressed on at least one anti-HER2 agent. Because this study started early from the year 2016 to 2019, many new drugs like CDK4/6 inhibitors, T-DM1 and pembrolizumab did not launch in China or were not covered by medical insurance at that time, so nearly all of the patients enrolled in this study did not receive today’s standard treatment due to drug accessibility and/or expensive cost but they failed standard treatment at that time.

The ORR was 25%, median PFS and OS was 4.8 and 15.4 months, respectively. These results were nearly consistent with those of previous clinical trials. Meanwhile, our study obtained a very favorable DCR of 86.5% which was higher than that was reported in most of trials.

A number of recent studies have explored the efficacy of apatinib, both alone and in combination, in pretreated MBC. Median PFS for apatinib monotherapy ranged 3.3–4.6 months, and that for the combination of apatinib and chemotherapy ranged 4.4–6.9 months(reviewed in **Table Supplement**). Median OS in these studies ranged 8.3–20.0 months (15, 16, 24, 27–31).We have reported a prospective multi-center phase II study of apatinib single or combination with endocrine therapy in HER2 negative breast cancer involving chest wall metastasis, the median PFS was 4.9 (95% CI: 2.1−8.3) months (29). Most of these trials were single-armed studies without control group. Only one retrospective study compared apatinib combined with capecitabine to capecitabine alone as the third-line therapy in advanced TNBC. The combination group had longer PFS (5.5 months vs. 3.5 months, p= 0.001) and a higher ORR (40.9% vs. 13.4%, p = 0.042) than the capecitabine group. As reported in the ASCENT study, median PFS was 5.6 months for sacituzumab govitecan (SG) and 1.7 months for chemotherapy in patients with heavily pretreated metastatic TNBC (32). Our subgroup analysis of patients with TNBC revealed a PFS of 3.7 months for apatinib combined with chemotherapy, however, due to the difference of study design and different enrolled population of studies and other limits, the results of different studies could not be directly compared. Further controlled research is needed to explore superiority of chemotherapy alone or in combination with apatinib.

Notably, although the current study was single-armed without control group, we conducted PFS analysis of the previous line of treatment in the same cohort, and median PFS was only 2.1 months, which was significantly shorter than that of apatinib–chemotherapy combination treatment. The relatively short PFS indicated the aggressiveness of the disease; hence, patients receiving apatinib–chemotherapy combination treatment had a heavier tumor burden and worse condition. Therefore, although it may be somewhat affected by the limitations of the self‐controlled case series method, this finding is still valuable. Additionally, some patients in our study received apatinib plus chemotherapy after a subpar response to initial chemotherapy alone, and a better response was observed in most patients, suggesting that the addition of apatinib can improve the efficacy of chemotherapy.

Another question is whether the addition of chemotherapy to apatinib produces better outcomes than apatinib alone. No clinical trials have directly compared apatinib monotherapy with the combination of apatinib and chemotherapy in breast cancer. Median PFS of apatinib monotherapy reported in clinical trials of breast cancer ranged from 3.3 to 4.6 months, which appeared inferior to the reported PFS of apatinib combination therapy (4.4–6.9 months). Bevacizumab monotherapy provided little clinical benefit in previously treated metastatic colorectal cancer. The median PFS and ORR for bevacizumab alone were 2.7 months and 3.3%, respectively, those for chemotherapy alone were 4.7 months and 8.6%, respectively, and those for the combination of bevacizumab and chemotherapy were 7.3 months and 22.7%, respectively (33). However, as a single agent, apatinib provided remarkable survival benefits in the third-line treatment of gastric cancer versus placebo (14).The exact reason is unclear, but in terms of the mechanism, bevacizumab is a monoclonal antibody directed against VEGFA that acts by binding and neutralizing all VEGFA isoforms (6). It is different that small-molecule TKIs block downstream signaling pathways by inhibiting the activity of VEGF receptors instead of binding to VEGF directly (6). More importantly, apatinib selectively inhibits VEGFR2, which is the key signaling receptor involved in the VEGF pathway. Compared with bevacizumab, apatinib has the advantage of oral bioavailability. Subgroup analysis of one small retrospective study (27 patients) revealed that PFS was even shorter in the apatinib combination group (20 patients, 3.1 months) than in the single-agent apatinib group (7 patients, 3.46 months) (34).

Moreover more studies on apatinib have focused on TNBC because of the limited treatment options for breast cancer of this subtype. Our study also included HER2-positive and ER positive breast cancer.These patients all failed previous available standard treatments. The subgroup analysis of our study suggested that there was no significant difference in PFS and ORR between different subtypes, results seemed to be better in the HER2-positive and hormone receptor-positive groups than in the TNBC group. However, the number of cases in HER2 positive group was too small, and some of them also received anti-HER2 targeted therapy in addition to apatinib and chemotherapy. In fact, it has been demonstrated that HER2 can increase VEGF protein synthesis by activation of the mTOR/p70S6K cap-dependent translation pathway in human breast cancer cells (35). VEGF might contribute to the aggressiveness of HER2-positive breast cancer (36). Additionally, some studies have suggested that the VEGF pathway could play a role in tamoxifen resistance in ER-positive breast cancer (37). These findings provide some theoretical basis for the effectiveness of apatinib in refractory HER2-positive and ER-positive breast cancers. In addition, subgroup analysis of our study revealed that median PFS for apatinib combined with chemotherapy was similar in different treatment lines, despite no patients obtained partial response in the first line treatment while 18.8% and 32.3% patients got partial response in the second and later lines. This may due to that the sample size for the first line group was too small(only five patients), and the size of their tumor lesions did not meet the measurable criteria, so it could only be evaluated as SD not PR even if the tumor was reduced significantly. Overall, our study suggested that the combination of apatinib and chemotherapy could be a potential treatment option in heavily pretreated MBC regardless of molecular subtypes and treatment lines.

In addition, new combinational regimens containing apatinib in MBC are under exploration (38–40). A phase II study reported the ORR (43.3%) and median PFS (3.7 months) of a combination of the immune checkpoint inhibitor camrelizumab and apatinib (250 mg) in patients with TNBC who received fewer than three lines of systemic therapy regardless of the line of therapy and the PD-L1 status (38). Another phase II study reported a favorable ORR (37.7%) and median PFS (8.1 months) for camrelizumab combined with apatinib (250 mg) and eribulin in heavily pretreated patients with advanced TNBC, and the PD-L1 status was not associated with ORR/PFS (39). And for germline BRCA1/2 mutated HER2 negative MBC, OlympiAD study and other studies have confirmed statistically significant PFS benefit of poly(ADP ribose) polymerase (PARP) inhibitors compared with chemotherapy treatment (40, 41). A study of fluzoparib (one PARP inhibitor) ± apatinib versus chemotherapy of physician’s choice in patients with HER2-negative MBC and germline BRCA mutations is ongoing (*NCT number:* ***NCT04296370*)**.

Concerning safety, owing to the high incidence of hypertension and hand-foot syndrome for high-dose apatinib in previous studies and in real-world clinical practice, in this current study, all patients received apatinib at a dose of 250 mg in combination with chemotherapy. This lower dose resulted in a lower incidence of AEs with comparable efficacy to high-dose apatinib as reported in previous studies. It is suggested that 250 mg might be the appropriate dose of apatinib when used in combination with chemotherapy, especially in patients who have received multi-line treatments.

In summary, the findings of our present study add to the existing knowledge for apatinib in MBC, it is possible to provide a basis for the treatment of refractory breast cancer patients with apatinib 250 mg combined with chemotherapy.

Nevertheless, there are several limitations in this study. Firstly, retrospective study design:the study relied on a retrospective review of medical records, which means that the data collected may not have been as comprehensive as it would have been in a prospective study. Retrospective studies are limited by the quality and completeness of the medical records, which could have led to missing or incomplete data on the treatment toxicity and the tumor assessment as well. Secondly, the study was lack of control groups, it only included patients who were treated with apatinib combined with chemotherapy, which makes it difficult to determine the extent to which the observed outcomes were due to the apatinib versus chemotherapy. Thirdly, the study only included patients from one institution, which may not be representative of the broader population of breast cancer patients; And the sample size was small, which lead to that it was insufficient to draw conclusions of subgroup analysis, the inferences about the results of subgroup analysis should be cautious.

Further multi-center, prospective and large randomized controlled trials are warranted to directly compare apatinib alone, chemotherapy alone, and combination of apatinib with chemotherapy to clarify the role of apatinib in advanced breast cancer treatment aiming to address these limitations.

## Data availability statement

The original contributions presented in the study are included in the article/**Supplementary Material**. Further inquiries can be directed to the corresponding authors.

## Ethics statement

The studies involving human participants were reviewed and approved by Ethics Committee of Peking University Cancer Hospital. The patients/participants provided their written informed consent to participate in this study.

## Author contributions

Study conception and design: RZ, GS, HL. Acquisition of data: RZ, YC, XRL, XG, AZ, HJ, BS, XL, YY, JZ. Drafting of the article: RZ, YC. Data analysis and interpretation: RZ, YC, XRL. All authors contributed to the work and approved it for publication.
