# Supplementary material for: Efficacy of apatinib 250 mg combined with chemotherapy in patients with pretreated advanced breast cancer in a real-world setting
Source: Front Oncol. 2023 Jun 16;13:1076469. doi: 10.3389/fonc.2023.1076469 (PMC10314217; doi:10.3389/fonc.2023.1076469)
Supplement: Supplementary file 1 [file Table_1.docx]

Table supplement. Clinical studies on treatment with apatinib in patients with metastatic breast cancer

| Study | Design | Patient population | Treatment arms | ORR | Median PFS（months） | Median OS(months) |
| --- | --- | --- | --- | --- | --- | --- |
| Hu et al Int.J.Cancer 2014 | prospective,open label, phase II, single arm | heavily pretreated mTNBC  IIa:N=25  IIb: N=59 | Apatinib monotherapy  750（IIa） or 500(IIb) mg/day | 10.7 | IIa: 4.6  IIb: 3.3 | IIa: 8.3  IIb: 10.6 |
| Hu et al. BMC Cancer 2014 | prospective,open label, phase II, single arm | Non-TNBC  2nd-5th line  N=38 | Apatinib monotherapy  500mg/day | 16.7 | 4.0 | 10.3 |
| Li et al. Medicine 2018 | Retrospective,controlled | Third line mTNBC  N=44 | apatinib 500mg+ capecitabine;  capecitabine | 40.9 vs 13.4  P=0.042 | 5.5 vs 3.5  P=0.001 | / |
| Zhu et al  ONCOLOGY LETTERS  2019 | observational study | previously  treated MBC  N=85 | Apatinib 450/500mg combine with chemotherapy | 23.2 | 4.4 | 11.3 |
| Liu et al  Front. Oncol.  2021 | Retrospective, real world data | Pretreated With Multiline Treatment MBC N=66 | Apatinib 850mg-250mg combine with or without chemotherapy | No ORR data.  CBR 40.9 | 6.0 | 10.0 |
| Li et al. Chin J Cancer Res 2021 | Prospective, phase II, single arm | HER2-negative MBC involving  chest wall metastasis  N=26 | Apatinib 500mg/250mg single or combine with endocrine therapy | 42.3 | 4.9 | 18.0 |
| Hu et al. Front. Oncol.2021 | Prospective, phase II, single arm | Heavily Pre-Treated MBC  N=31 | apatinib 500/450mg+etoposide capsules | 35.5 | 6.9 | 20.4 |
| Zhu et.al  Cancer Biol Med 2021 | Prospective, phase II, single arm | heavily pretreated HER2-negative MBC  N=40 | apatinib500/425 mg daily plus oral vinorelbine 60mg/m2 every week | 17.1 | 5.2 | 17.4 |
